# RNA Virus Replication Complexes

**DOI:** 10.1371/journal.ppat.1000943

**Published:** 2010-07-22

**Authors:** Yizhi Jane Tao, Qiaozhen Ye

**Affiliations:** Department of Biochemistry and Cell Biology, Rice University, Houston, Texas, United States of America; University of California San Francisco, United States of America

The majority of viruses infecting animals and plants today are RNA viruses [1]. There are double-stranded (ds) RNA viruses with dsRNA genomes, as well as (+) and (−)RNA viruses whose genomes are single-stranded (ss) RNA of either positive or negative polarity. RNA viruses have small genomes that rarely exceed 30 kb in size, and a large portion of their genomes is used to encode proteins involved in viral RNA replication. Viral RNA synthesis is catalyzed by the virally encoded RNA-dependent RNA polymerase (RdRp), which lacks any proofreading activity. Although the details of RNA replication greatly vary among viruses, common principles clearly exist in the organization of the replication machinery of different RNA viruses.

## (+)RNA Viruses Replicate Their Genomes on the Surface of Host Membranes

For many (+)RNA viruses, RNA replication requires viral enzymes such as RdRp, helicase, capping enzymes, and NTPase, as well as non-enzymatic proteins that participate in the assembly of the viral replication complex. Host cell proteins often play essential roles in (+)RNA virus replication as well. (+)RNA virus replication is asymmetric: the synthesis of (+)RNA, the one that is packaged into the progeny virus, is up to ∼100 times greater than that of (−)RNA [1]. Both the structure of the replicase itself and the *cis*-acting signals in the viral RNA template have been implicated in regulating the relative levels of genomic and antigenomic RNAs.

Host membranes play an important role in (+)RNA virus replication, as the RNA replication complexes released from membranes during purification generally lose the ability to catalyze true RNA replication, although a limited activity may be retained. In general, the intracellular membranes of cells infected with (+)RNA viruses rearrange to form anchor sites for viral RNA replication complexes [2]. Membrane tethering of the replication complexes is facilitated by non-structural proteins with hydrophobic sequences that enable membrane integration or interaction. In picornavirus-infected cells, viral RNA replication occurs on the cytoplasmic surfaces of double-membrane vesicles derived from the endoplasmic reticulum (ER) [3]. Two-dimensional (2D) arrays of polymerase-containing oligomeric structures have been observed on the surface of these vesicles, and the structural integrity of these 2D arrays correlates with the cooperative RNA binding and RNA elongation activities of the polymerase [4]. In cells infected with the flock house virus, outer mitochondrial membranes invaginate to create open-necked spherules of ∼50 nm in size [5]. On average, each spherule contains ∼100 membrane-spanning, self-interacting protein A molecules (i.e., the multifunctional RNA replication factor) and two to four genomic RNA replication intermediates [5]. Membranes may function to expedite the assembly of replication complexes, to protect/sequester viral RNAs, and also to help segregate the products from templates during replication.

**Figure 1:**
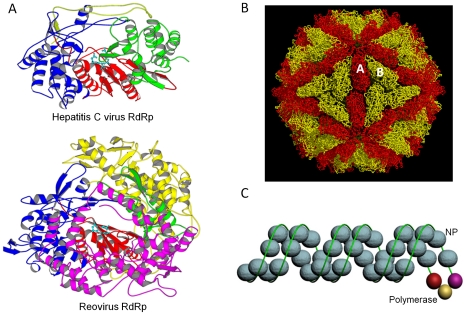
RNA virus replication machineries. (A) RdRps of hepatitis C virus and reovirus. Hepatitis C virus is a (+)RNA virus from the Flaviviridae family, while reovirus is a dsRNA virus belonging to the Reoviridae family. In both structures, the fingers, palm, and the thumb of the polymerase are colored in blue, red, and green, respectively. Yellow and magenta highlight the N- and C-terminal domains, respectively. The three aspartic acid residues in the polymerase catalytic active site are shown in cyan. Reovirus RdRP has large N- and C-terminal domains with distinct functions: the N-terminal domain maintains the closed conformation of the active site, and the C-terminal domain serves as a processivity factor like a sliding clamp. (B) The yeast L-A virus (Totiviridae family). The two independent sets of capsid protein molecules, 60 copies each, are shown in red and yellow, respectively. The viral RdRP is likely to be tethered to the inner capsid near the 5-fold symmetry axis, and viral transcripts made inside the core are released through channels on or near the 5-fold axes. (C) An influenza A virus (Orthomyxoviridae family) RNP model. Blue spheres represent NP monomers, and the green line shows vRNA. A short duplex formed between the 5′ and the 3′ ends provides the binding site for the heterotrimeric RdRp. Overall, the RNP assumes a rod-shaped, double-helical structure that remains intact even after the vRNA is removed.

Although little is known about the structure of the membrane-associated RNA replication complexes, (+)RNA virus RdRps have been well characterized to have an overall right-handed shape with three domains, the fingers, palm, and the thumb (reviewed in [6]) (Figure 1A). The palm domain contains an invariant, central, four-stranded β-sheet, with five highly conserved motifs named A to E. Motifs A and C, which contain the sequence –DxxxxD– and –GDD–, respectively, play important roles in metal ion coordination and nucleotide substrate selection during catalysis. The fingers and thumb domains are located on opposite sides of the active site canyon. Most (+)RNA virus RdRps can initiate RNA synthesis de novo, but a number of them use viral protein primers (VPgs) and produce protein-linked genomes. In the RdRp from the foot-and-mouth disease virus, uridylated VPg occupies a site similar to the typical RNA primer in the active site canyon, with the key residue Tyr3 and its associated uridylate (UMP) projected into the active site [7].

## dsRNA Viruses Replicate and Transcribe Their Genomes in Intact Core Particles

Genome replication by dsRNA viruses occurs in subviral particles. These subviral particles, also called the cores, have an intact viral capsid that encloses the viral genome and RdRp molecules. For complex, multi-layered dsRNA viruses, the core is derived from the virion by removing outer capsid proteins during entry. Simple dsRNA viruses without extracellular life cycles often do not have additional capsid layers beyond the core. Progeny cores are assembled from mRNAs, which are then replicated inside the particle to generate the dsRNA genome. The number of gene segments can vary from one to 12 for different dsRNA viruses.

To date, all seven families of encapsidated dsRNA viruses have been analyzed by cryo-EM or crystallography (Figure 1B) (see [8]–[14] for crystal structures). Except for the birnavirus, dsRNA viruses share structural similarity by having a characteristic, 120-subunit T = 1 capsid surrounding their genome. In viruses with high genomic RNA densities, the genome is packed into liquid crystalline arrays that give rise to concentric shells of density, 25–30 Å apart, in the particle interiors. Due to the lack of icosahedral symmetry, viral polymerase is invisible in the structure of the viruses. Reoviruses have ∼12 copies of the polymerase in each virion, each associated with one of the ten to 12 dsRNA gene segments that make up the viral genome. The polymerases are likely to be tethered to the capsid near 5-fold symmetry axes through noncovalent interactions [15]. In addition to the central polymerase domain, dsRNA virus RdRps often have elaborate N- and C-terminal domains that keep the polymerase in the closed conformation (Figure 1A). In particular, the Reoviridae polymerase forms a rigid, caged structure with four well-defined tunnels that allow the inward and outward trafficking of the template, substrates, and products [16]. Capsid proteins play a key role in regulating polymerase function, as the polymerase alone is either inactive or exhibits only limited activities. This intra-particle mechanism of RNA synthesis coordinates genome packaging with replication during the infectious cycle.

A key question about dsRNA virus replication is how the (−)RNA template is selected from the dsRNA genome during transcription. A 5′-cap binding site, identified on the reovirus polymerase, provides a neat solution for some dsRNA viruses of high eukaryotic hosts [16]. Because only the (+)RNA strand is capped, cap binding keeps the polymerase proximal to the 3′-end of the (−)RNA strand, thus allowing efficient initiation of transcription. Negative charges on the inner surface of dsRNA viral capsids may help to facilitate viral genome movement during transcription. Viral transcripts are likely to exit the core through pores on or near the 5-fold symmetry axes.

## (−)RNA Viruses Replicate Using Protein-Coated Templates and Generate Protein-Stabilized, Single-Stranded RNAs

(−)RNA viruses have a helical-shaped, protein-coated genome, also known as the ribonucloeprotein complex (RNP), which functions as the sole template for viral RNA replication and transcription (Figure 1C). Newly made viral RNA (vRNA) and the replication intermediate (cRNA) are packaged as RNPs and stabilized in a single-stranded form immediately after their synthesis.

The composition of the RNA replication machinery differs between segmented and non-segmented (−)RNA viruses. The influenza A virus, a segmented (−)RNA virus, contains eight vRNAs, each packaged as a double helical-shaped RNP with multiple nucleoproteins (NPs) and a heterotrimeric polymerase (PA, PB1, and PB2) that is bound to the conserved vRNA termini (Figure 1C). Each NP molecule binds to a 24-nt RNA at the outer periphery of the NP scaffold [17]. The influenza A virus polymerase has a compact structure [17] with at least four active sites: the polymerase active site and a 5′ vRNA binding site in PB1, an endonuclease active site in PA [18], [19], and a cap binding site in PB2 [20]. During transcription, the polymerase uses a short capped RNA fragment snatched from cellular mRNAs to initiate RNA synthesis. To switch from transcription to replication mode, it is necessary to change from capped RNA-primed initiation to unprimed initiation and to prevent pre-mature termination and polyadenylation. Free NP is essential for viral RNA replication, possibly because (1) NP interacts with the polymerase, thus altering its activity [21], or (2) NP prevents the degradation of the cRNA intermediate by forming RNP structures [22]. It has been proposed that viral RNA transcription is catalyzed by the polymerase associated with the vRNP template, whereas RNA replication is catalyzed by a newly made polymerase molecule [23]. The specific binding of the viral polymerase to the 5′-end of newly synthesized vRNAs presumably helps to recruit additional NPs, thus ensuring specific encapsidation of vRNA.

RNA replication of non-segmented (-)RNA viruses, such as the rabies virus and the vesicular stomatitis virus (VSV), requires an intricate interplay of the nucleoprotein N, the large protein L, a non-enzymatic P, and the N-enwrapped genome, also called the nucleocapsid [24]. In the nucleocapsid, each N protein has two lobes angled together to form a cavity that accommodates seven or nine bases of RNA [25]–[27]. In addition to nucleotide polymerization, L also possesses enzymatic activities for 5′ cap synthesis. During RNA synthesis, P delivers L to the active template through an N-P interaction that involves two adjacent N proteins in the nucleocapsid [28]. L-P binding to the N-RNA template triggers conformational changes in the nucleocapsid and allows the polymerase access to RNA.

## Summary

Different replication strategies are used by (+), (−), and dsRNA viruses to limit dsRNA exposure, to regulate (+)RNA versus (−)RNA synthesis, and to modulate transcription versus replication throughout the infection cycle. The differences in the functionalities of the replication complexes are reflected in their structural composition, their subcellular localization, and in the interaction of the complex with viral RNA templates. It is expected that studies of RNA virus replication machineries will have a large impact on antiviral drug development due to their specific activities in virus replication.
